# Development of Open-Tubular-Type Micro Gas Chromatography Column with Bump Structures

**DOI:** 10.3390/s19173706

**Published:** 2019-08-26

**Authors:** Janghyeon Lee, Si-Hyung Lim

**Affiliations:** 1 Department of Mechanics and Design, Kookmin University, Seoul 02707, Korea; 2 School of Mechanical Engineering, Kookmin University, Seoul 02707, Korea

**Keywords:** volatile organic compounds, gas chromatography, MEMS, separation column, bump structures

## Abstract

Gas chromatography (GC) is the chemical analysis technique most widely used to separate and identify gas components, and it has been extensively applied in various gas analysis fields such as non-invasive medical diagnoses, indoor air quality monitoring, and outdoor environmental monitoring. Micro-electro-mechanical systems (MEMS)-based GC columns are essential for miniaturizing an integrated gas analysis system (Micro GC system). This study reports an open-tubular-type micro GC (μ-GC) column with internal bump structures (bump structure μ-GC column) that substantially increase the interaction between the gas mixture and a stationary phase. The developed bump structure μ-GC column, which was fabricated on a 2 cm × 2 cm μ-GC chip and coated with a non-polar stationary phase, is 1.5 m-long, 150 μm-wide, and 400 μm-deep. It has an internal microfluidic channel in which the bumps, which are 150 μm diameter half-circles, are alternatingly disposed to face each other on the surface of the microchannel. The fabricated bump structure μ-GC column yielded a height-equivalent-to-a-theoretical-plate (HETP) of 0.009 cm (11,110 plates/m) at an optimal carrier gas velocity of 17 cm/s. The mechanically robust bump structure μ-GC column proposed in this study achieved higher separation efficiency than a commercially available GC column and a typical μ-GC column with internal post structures classified as a semi-packed-type column. The experimental results demonstrate that the developed bump structure μ-GC column can separate a gas mixture completely, with excellent separation resolution for formaldehyde, benzene, toluene, ethylbenzene, and xylene mixture, under programmed operating temperatures.

## 1. Introduction

The analysis of volatile organic compounds (VOCs) is fundamental in a wide range of applications, including public safety management, non-invasive medical diagnoses, indoor air quality monitoring, and outdoor environmental monitoring [1,2,3,4,5,6,7,8,9]. Although VOCs can be collectively detected by various gas sensors and detectors, these devices typically lack the selectivity to discriminate between constituents of complex mixtures. Simple gas compounds can be analyzed by high-performance mass spectrometry and infrared spectroscopy systems, whereas complex mixtures typically require a gas chromatography (GC) system [10,11,12]. GC techniques are beneficial and reliable in the analysis of complicated mixture gases in the aforementioned applications. Such analyses are usually performed with commercial bench-top instruments such as a gas chromatography–mass spectrometer or a gas chromatograph equipped with a flame ionization detector (FID) [13,14]. Therefore, a GC system that consists primarily of a preconcentrator and separation column is required to improve the sensitivity and selectivity of the detector, and such systems are used for the determination of the discrete components in complex mixtures. However, currently available GC system instruments are generally bulky, expensive, time-consuming to operate, consume large amounts of energy, and require highly trained technicians [15,16].

Miniaturization of gas chromatographic systems provides numerous advantages. A typical GC system with reduced size affects the kinetics of the exchanges by decreasing the distances the solutes must diffuse during mass transfer between the mobile and stationary phases [17,18]. Since the late 1970s, various micro-gas chromatography (μ-GC) systems have been reported, and a subset of the necessary components of these systems has been lithographically microfabricated [19]. The resultant highly miniaturized systems based on micro-electromechanical systems (MEMS) have been used to fabricate GC columns [20,21,22,23]. However, most of the μ-GC systems reported to date have involved manual assembly of separately fabricated μ-GC components using commercially available off-chip fluidic interconnects. Furthermore, separating various gases remains difficult because of the limited device size [24,25,26,27,28,29].

**Figure 1:**
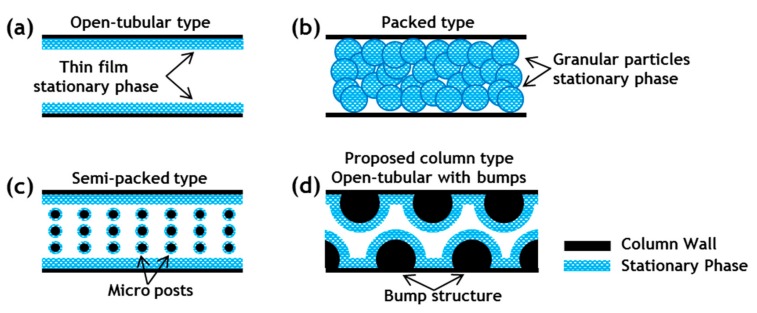
Various gas chromatography (GC) column types. (**a**) Open-tubular-type column with a thin-film stationary phase coated onto the inner wall of the column; (**b**) packed-type column in which the stationary phase of the small granular particles is packed inside the column; (**c**) semi-packed-type column featuring a micro-post array fabricated along the length of the column channel through a micromachining process. Typically, semi-packed columns are coated with a thin-film stationary phase; (**d**) proposed bump structure micro GC (μ-GC) column. The proposed column is coated with a thin-film stationary phase and is mechanically robust, demonstrating high-performance gas separation.

μ-GC columns are generally classified into open-tubular and packed types, as shown in Figure 1a,b. The stationary phase of the open-tubular column is coated onto the inner wall of the column channel in thin-film form, whereas in the packed column, the stationary phase is packed with small granular particles inside the column channel. The choice between an open-tubular-type column and a packed-type column involves a tradeoff between sample capacity and separation efficiency. Open-tubular-type columns lack large sample capacity because of their relatively low surface area. Packed-type columns have a greater surface area, thereby providing greater sample capacity than the open-tubular-type columns. However, the greater surface area of the packed-type columns produces greater pressure drops and greater eddy diffusion, thereby reducing the separation efficiency [30].

μ-GC columns that feature a micro-post array along the length of the column channel, referred to as semi-packed-type columns (Figure 1c) have recently been reported to enhance the separation efficiency and increase the sample capacity [20,31,32,33]. These semi-packed-type columns can attain greater separation efficiency and sample capacity than conventional open-tubular-type columns because of their reduced width and increased surface area. Furthermore, because of the symmetrical distribution resulting from the post array being embedded in the channel, the semi-packed-type columns have lower pressure drops and diminished eddy diffusion effects compared with packed-type columns. As a result of these advantages, numerous studies on semi-packed-type μ-GC columns have focused on improving the separation efficiency through design of a column with a high aspect ratio micro-post array. However, the micro-post array with a high aspect ratio in the column channel can easily collapse and distort because of adhesive forces and capillary forces during the etching, drying, and bonding processes involved in the microfabrication processes [34,35,36,37,38,39,40,41,42,43]. In addition, traditional bonding methods such as anodic bonding and fusion bonding for sealing the μ-GC column channel may fail if step height occurs between each post with a high aspect ratio during the process [44]. These defects such as collapse and distortion of the micro-post array pattern because a high aspect ratio in the columns can cause pooling of the stationary phase and channel clogging at the defect area [45,46,47,48]. In most cases, the micro-posts are exposed to the liquid during coating of the stationary phase. Generally, the torque generated by the capillary force is greater than the adhesion force between the micro-post and the substrate. Therefore, even if a micro-post array with a high aspect ratio has been well fabricated without defects, the capillary force could easily induce lateral collapse as the liquid evaporates from the surface of the micro-posts [49,50,51,52].

In this paper, we demonstrate a mechanically robust bump structure μ-GC column with a comparable separation performance. Figure 1d shows a schematic of the proposed open-tubular μ-GC column with internal bump structures (bump structure μ-GC column). The bump structure μ-GC column with dimensions of 2 cm × 2 cm was designed and fabricated through the MEMS process. To increase the interaction between the gas molecules and the stationary phase, alternating bump structures were designed on both walls of the channel. The non-polar stationary phase was coated onto the inner wall of the column. The μ-GC module was completed by mounting the μ-GC column onto a printed circuit board (PCB) for column temperature control. Based on the Golay equation, the separation efficiency of the developed bump structure μ-GC column was compared with a standard open-tubular-type commercially available GC column (commercial GC column) and a typical μ-GC column with internal post structures (post structure μ-GC column) classified as a semi-packed-type column. In addition, the separation performance tests were conducted for a mixture of five gases—formaldehyde (F), benzene (B), toluene (T), ethylbenzene (E), and xylene (X)—chosen as representative hazardous VOCs.

## 2. Experimental Section

### 2.1. Fabrication of Micro-GC Column Module

#### 2.1.1. MEMS Process of Micro-GC Chip

**Figure 2:**
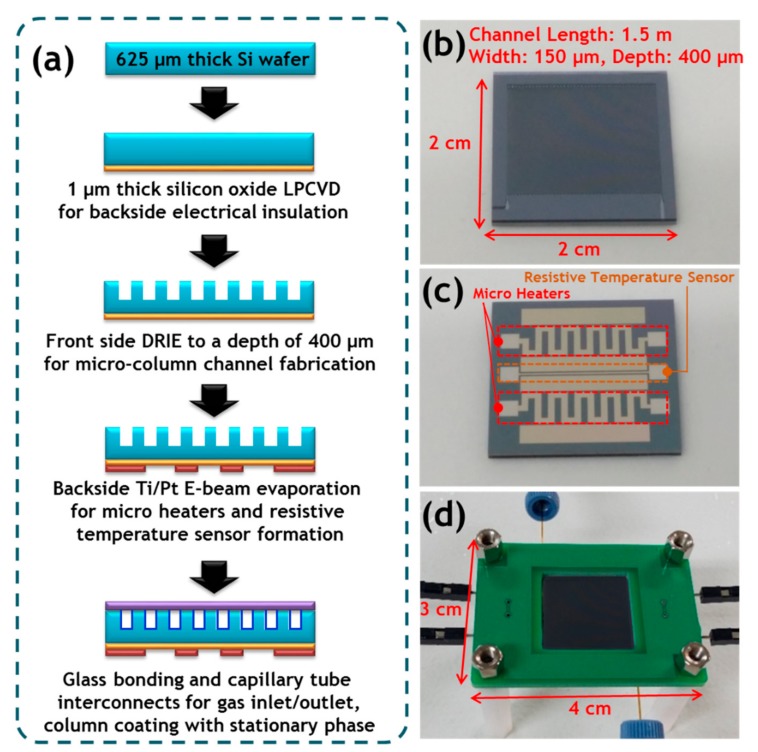
Fabrication of the bump structure μ-GC module. (**a**) Fabrication process flow of the bump structure μ-GC column; (**b**) front side of the chip showing the bump structure μ-GC column; (**c**) back side of the chip showing patterned platinum micro-heaters and resistive temperature sensor (RTS) for temperature control of the μ-GC column; (**d**) packaged μ-GC module with the fused-silica capillary tube, micro-capillary adapters, etc. (size: 3 cm × 4 cm).

The μ-GC column was fabricated through the MEMS processes. Figure 2 shows the fabrication process and photographs of the μ-GC column.

The fabrication flow of the bump structure μ-GC column is shown in Figure 2a. The procedure was initiated with a standard six-inch silicon wafer with both sides polished and a thickness of 625 μm. The serpentine-shaped column was defined through a photolithographic process and etched onto the front side of the substrate via deep reactive-ion etching based on the standard Bosch process. This etching process formed the serpentine-shaped column channel and the micro-bumps (which have a radius of 75 μm) along the fluidic path to increase the interaction surface area between a stationary phase and a mobile phase. It has an internal microfluidic channel in which the bumps are alternatingly disposed to face each other at 150 μm intervals on the surface of the micro-column channel. The formed bump structures are mechanically robust because they are an integrated body with a silicon substrate along with the column channel. The microfabricated column was 1.5 m long with channel width and depth of 150 and 400 μm, respectively, as shown in Figure 2b. Then, the micro-heaters and the resistive temperature sensor (RTS) for temperature ramping and monitoring, respectively, were constructed as a 20 nm/200 nm Ti/Pt stack deposited by electron beam (E-beam) evaporation and patterned by the lift-off process on the backside of the silicon substrate, as shown in Figure 2c. The resistances of the micro-heater and the RTS were 43 and 38 Ω, respectively. The open surface of the wafer was anodically bonded to a Pyrex 7740 glass wafer to seal the channel in intimate contact, and a high electric field of 900 V was applied at 380 °C for 30 min. Next, the wafer was diced into individual columns with a size of 2 cm square and the fused-silica capillary tubes with 360 μm O.D. and 150 μm I.D. were connected to the microfluidic in/out ports of the column using epoxy (Duralco 4703, Contronics Corp., Brooklyn, NY, USA). The μ-GC chip was combined with a PCB for temperature control and micro-capillary adapters (P-770, IDEX Corp., Lake Forest, IL, USA) for connection to the gas supply and detector. Figure 2d shows the fabricated μ-GC module with dimensions of 4 cm (L) × 3 cm (W).

For comparison of the separation performance, the post structure μ-GC column was fabricated through the same micromachining processes previously described. The circular post structures formed in the semi-packed-type column channel exhibited a diameter of 30 μm and were disposed at intervals of 150 μm along the microchannel.

The MEMS-based GC columns were commonly fabricated on 2 cm × 2 cm μ-GC chips. The column channels were 1.5 m-long, 150 μm-wide, and 400 μm-deep and were coated with a non-polar stationary phase in the form of a thin-film.

#### 2.1.2. Stationary Phase Coating of Column Channel

The stationary phase is the key component of the GC column. It reacts with the analytes and affects the rate of the diffusion in and out of the mobile phase. The non-polar stationary phase used in this study primarily interacts with analytes by the van der Waals force. Analytes with higher molecular weights generally have a stronger interaction with the stationary phase and demonstrate longer retention times.

**Figure 3:**
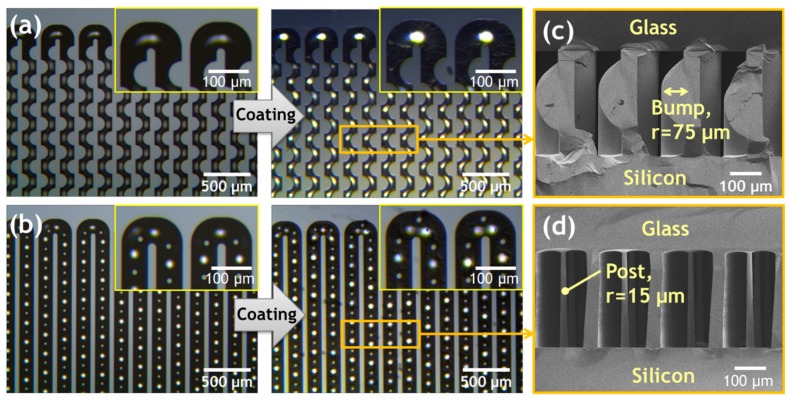
Top-view optical micrographs of the μ-GC columns before and after an OV-1 thin-film stationary phase was coated onto (**a**) the bump structure μ-GC column and (**b**) the post structure μ-GC column; (**c**) cross-sectional SEM image (tilted to show the bump structures formed in the column channel) of the bump structure μ-GC column; (**d**) cross-sectional SEM image of the post structure μ-GC column.

After the channel was sealed, the stationary phase was usually applied via the liquid coating method. Static coating was the most frequently used method, and a non-polar stationary phase was generally used for the separation column. In the static coating procedure, the GC column was filled with the stationary-phase solution; one end of the column was sealed, and the other end was connected to a vacuum pump. The solvent was slowly evaporated by the reduced pressure of the vacuum pump until all of the solvent was completely evaporated; the coating was then complete. The static coating allows a uniform distribution of films because of the lack of axial motion of the stationary phase during deposition [53]. The non-polar stationary phase (OV-1, Sigma-Aldrich, St. Louis, MO, USA) was used to coat the microfabricated column using the static coating procedure. The coating solution was prepared by completely dissolving 120 mg of OV-1 and 1 mg of dicumyl peroxide (Sigma-Aldrich) in 30 ml of a mixture of n-pentane and dichloromethane (1:1 volume ratio). The mixture was agitated with a vortex mixer (Vortex-Genie 2, Scientific Industries Inc., Bohemia, NY, USA) for 30 min to ensure full dissolution. The outlet of the column was connected to a miniature vacuum pump (SP-500 EC, Schwarzer Precision Corp., Essen, Germany), which was used to inject the coating solution into the column. After the column was filled with the prepared coating solution, the miniature vacuum pump was turned off and held for 5 min to ensure that the stationary phase had sufficient time to attach to the channel wall. The solvent was then evaporated by the vacuum pump until the column appeared empty from one end; the other end was sealed with a conventional GC septum. Subsequently, the coating chemicals were cross-linked to the inner surface of the column by heating at 150 °C for 2 h under flowing nitrogen in an electric furnace (KSL-1100X, MTI Corp., Richmond, CA, USA), leaving behind a film of thin and uniform stationary phase with a thickness of about 0.1~0.2 μm on the column wall [53]. The micrographs before and after the OV-1 thin-film stationary phase was coated onto the bump structure μ-GC column and the post structure μ-GC column are shown in Figure 3a,b. Figure 3c,d show the cross-sectional scanning electron microscopy (SEM) images of the bump and post structure μ-GC columns after anodic bonding.

### 2.2. Experimental Setup and Conditions for the Separation Performance

**Figure 4:**
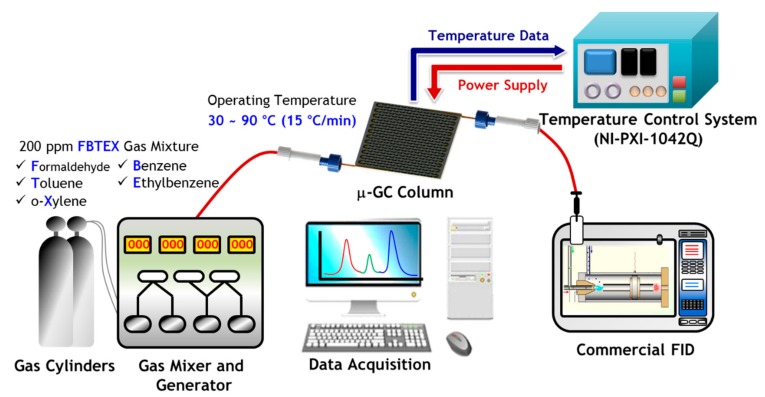
Schematic of the experimental setup used to test the separation performance of the GC columns. The sample amount of 50 μL of FBTEX mixture gas with a concentration of 200 ppm was injected into the μ-GC column by the gas mixer and generator, and nitrogen was used as the carrier gas at a flow rate ranging from 0.3 sccm to 0.4 sccm. The temperature of the μ-GC column was maintained at 30 °C for 1 min, ramped to 90 °C at a ramping rate of 15 °C/min, and then maintained at 90 °C for 1 min. The temperature programming of the column was performed by micro-heaters and RTS of the μ-GC column using the hardware control system. The gas components separated through the μ-GC column were detected by the commercial FID; the separation results are shown through the data acquisition system.

The experimental setup is illustrated in Figure 4. The separation performance tests of the fabricated μ-GC columns were performed for the FBTEX mixture gas known as representative hazardous VOC gases. The FBTEX mixture gas (RIGAS Co., Ltd., Daejeon, South Korea) with a concentration of 200 parts per million (ppm) was injected into the μ-GC column in an amount of 50 μm via the gas mixer and generator; nitrogen was used as a carrier gas at a constant flow rate using a mass flow controller. The micro-heaters and RTS of the μ-GC column were connected to a hardware control system (PXI-1042Q, National Instr., Austin, TX, USA) containing a power supply.

On the other hand, the temperature is an important factor that affects the separation performance of the column. In a GC column, there is an equilibrium of the analyte between the mobile phase and the stationary phase, which is affected by the column temperature. When the separation is performed under isothermal conditions at low temperatures, the weakly retained compounds are adequately separated while strongly retained compounds demonstrate severe tailing and long retention times. Conversely, a separation performed under higher isothermal conditions leads to excellent separation of strongly retained compounds but results in poor separation of weakly retained compounds. This general elution issues can be solved by programming the temperature of the GC column [54]. Therefore, in this study, the temperature of the columns was programmed to obtain an excellent separation chromatogram for the FBTEX mixture using the patterned micro-heaters and RTS on the backside of the μ-GC chip controlled by the hardware control system.

The temperature of the μ-GC column was maintained at 30 °C for 1 min and ramped to 90 °C at a ramping rate of 15 °C/min and maintained at 90 °C for 1 min. The electric power values from 1.1 W to 3.3 W were used for temperature ramping from 30 °C to 90 °C. The gas components separated through the μ-GC column were detected with a commercial FID (YL 6100 GC, Young Lin Instr., Anyang, South Korea), and the separation results were collected through a data acquisition system. Separation performance tests were carried out for both of the fabricated μ-GC columns under the same experimental conditions, and the results were compared. On the other hand, the commercial GC column with a bonded-porous-silica-based stationary phase (GS-Gaspro, Agilent, Santa Clara, CA, USA) was prepared by being cut to a length of 1.5 m (320 μm I.D.), which is the same length as the μ-GC columns, for the comparison of the gas separation efficiency based on the Golay equation. This commercial GC column is specified for the application to the separation of C_1_-C_12_ hydrocarbons as described in [55,56].

## 3. Results and Discussion

**Figure 5:**
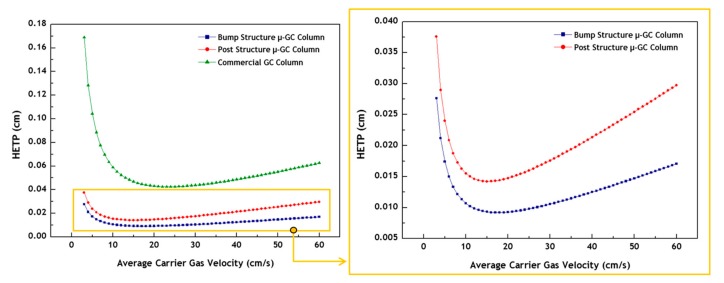
Height-equivalent-to-a-theoretical-plate (HETP) versus average carrier gas velocity for the commercial GC column and the μ-GC columns of bump and post structures.

The separation efficiency of a GC column can be defined by the height-equivalent-to-a-theoretical-plate (HETP), expressed in Equation (1) [57].

(1) $$HETP=\frac{2D_{g}}{ū}f_{1}f_{2}+\left[\frac{\left(1+9k+25.5k^{2}\right)}{105{\left(k+1\right)}^{2}}\frac{w^{2}f_{1}}{D_{g}f_{2}}+\frac{2}{3}\frac{k}{{\left(k+1\right)}^{2}}\frac{{\left(w+h\right)}^{2}d_{f}^{2}}{D_{s}h^{2}}\right]ū$$

where *D_g_* and *D_s_* are the binary diffusion coefficients in the mobile and stationary phases, respectively; *ū* is the velocity of the carrier gas; and *f*_1_ (varies between 1 and 1.125) and *f*_2_ (varies between zero and one) are the Gidding-Golay and Martin-James gas compression coefficients, respectively; *w* and *h* are the width and height of the column channel, respectively; *k* and *d_f_* are the retention factor and stationary phase thickness, respectively. The smaller the HETP value for a given column length, the higher the separation efficiency of the column. As a very thin thickness of the stationary phase film is assumed, the contribution of band-broadening due to diffusion in the stationary phase can be neglected. Therefore, the 3rd term in Equation (1) can be ignored to simplify the calculation [58]. An *n*-hexane was used to determine the average carrier gas velocity for calculating the corresponding HETP value of the GC columns. In addition, effective widths of 75 μm and 120 μm for the bump and post structure μ-GC columns, respectively, were used in the calculations. The value of *D_g_* was chosen as 0.076 cm^2^/s [59]. Figure 5 shows the calculated HETP versus the average carrier gas velocity. Based on the Golay equation for round cross-section columns, the commercial GC column has the HETP value of 0.043 cm (2,330 plates/m) at the optimal carrier gas velocity of 23 cm/s (inlet pressure of 2.3 psi). The bump and post structure μ-GC columns have the HETP values of 0.009 cm (11,110 plates/m) and 0.014 cm (7140 plates/m) at the optimal carrier gas velocities of 17 cm/s (inlet pressure of 5.5 psi) and 15 cm/s (inlet pressure of 4.1 psi), respectively, based on the Golay equation for rectangular cross-section columns. The bump structure μ-GC column showed about 1.6 times higher separation efficiency than the post structure μ-GC column in the comparison of HETP values, or plate numbers. The plate number of the bump structure μ-GC column was 11,110 plates/m, which is comparable to the reported plate number of 4200~12,800 plates/m in other recent studies related to MEMS-based columns with similar physical dimensions [33,60,61]. On the other hand, the separation efficiency of the commercial GC column was about five times lower than that of the bump structure μ-GC column. As a result of the low separation efficiency of the commercial GC column, it was excluded from the further separation performance test in this study.

**Figure 6:**
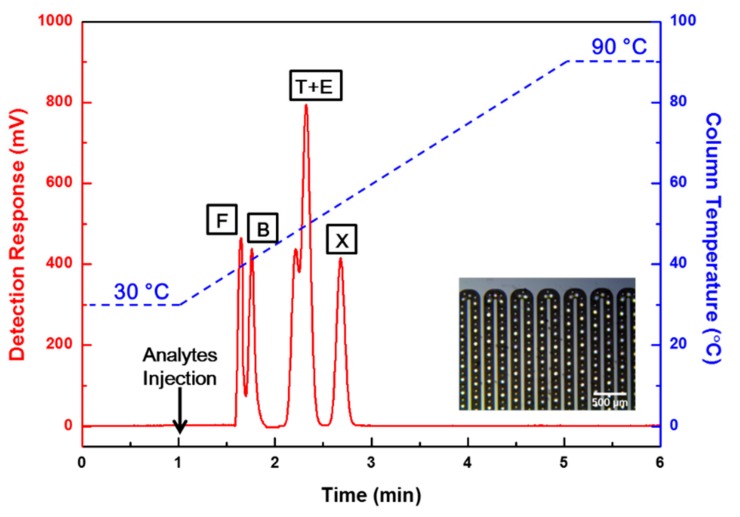
Chromatograms showing the separation results obtained through the post structure μ-GC column for the FBTEX mixture gas. The temperature of the μ-GC column was maintained at 30 °C for 1 min, ramped to 90 °C at a ramping rate of 15 °C/min, and then maintained at 90 °C for 1 min. The temperature programming of the column was performed by micro-heaters and an RTS of the μ-GC column. In the test, all five peaks were detected but two overlapped peaks appeared.

**Figure 7:**
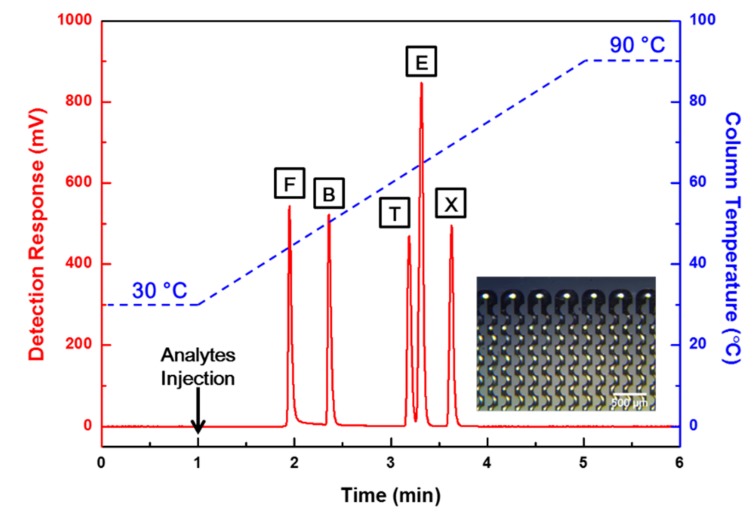
Chromatograms of the separation result obtained through the bump structure μ-GC column proposed in this study. The temperature of the μ-GC column was maintained at 30 °C for 1 min, ramped to 90 °C at a ramping rate of 15 °C/min, and then maintained at 90 °C for 1 min. The temperature programming of the column was performed by micro-heaters and RTS of the μ-GC column. Five different peaks were completely separated in the test.

Separation performance tests were conducted using the fabricated bump structure μ-GC column and post structure μ-GC column for the FBTEX mixture gas under the same analytical conditions. The commercial FID detected the gas components separated through the μ-GC columns, and the results were compared and analyzed. Figure 6 and Figure 7 show the separation results obtained with each column for the FBTEX mixture gas through the FID signal. The *X*-axis shows the time (min), the left *Y*-axis shows the FID detection signal level (mV), and the right *Y*-axis shows the column temperature (°C). The temperature of the column was maintained at 30 °C and 90 °C for 1 min, respectively, to stabilize the FID signal at the start and end of the analysis. The mixture sample of FBTEX was injected at the 1-min point after the beginning of the analysis. Subsequently, the gas separation was started with the injection of nitrogen, which is an inert gas and does not react with the FID, as a carrier gas at a flow rate ranging from 0.3 sccm to 0.4 sccm into the μ-GC column.

Figure 6 shows the results of the gas separation test for the post structure μ-GC column, all five peaks were detected. However, two overlapped peaks appeared because of lower separation resolutions. On the contrary, the bump structure μ-GC column showed excellent separation performance, with five completely separated peaks, as shown in Figure 7.

The separation resolution was also calculated as the separation performance index of the columns. The separation resolution (*R_s_*) is expressed in Equation (2) [15,16].

(2) $$R_{s}=\frac{{\left(t_{r}\right)}_{B}-{\left(t_{r}\right)}_{A}}{({\left(w_{b}\right)}_{A}+{\left(w_{b}\right)}_{B})/2}$$

where *t_r_* and *w_b_* are the retention time and the peak width at the base, respectively. The data for the retention time (*t_r_*) and peak width (*w_b_*) for the separated individual gas components presented in Figure 6 and Figure 7 are compiled in Table 1.

**Figure 8:**
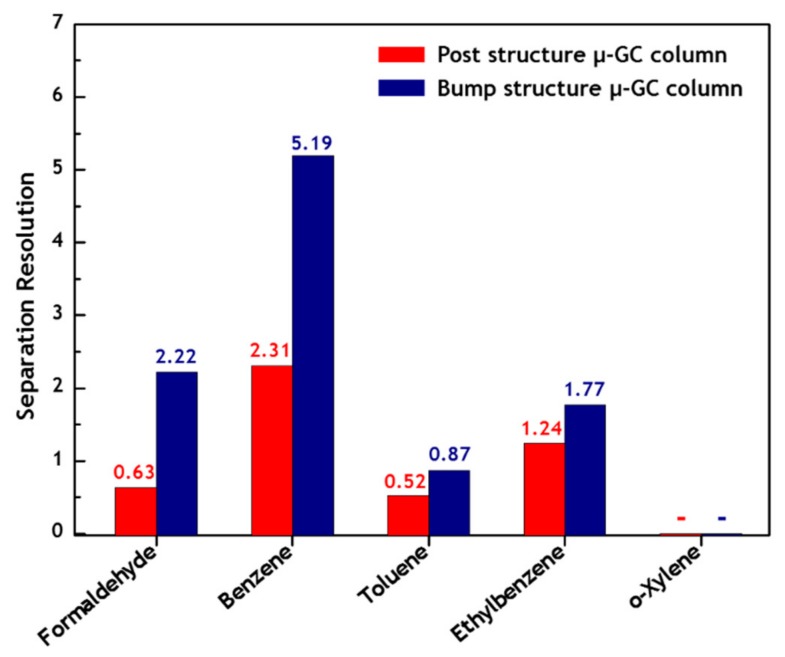
Peak resolutions (*R_s_*) calculated for formaldehyde, benzene, toluene, ethylbenzene, and *o*-xylene gases based on the separation results through the μ-GC columns.

Figure 8 shows the calculated *R_s_* values for the F, B, T, E, and X gases based on the separation results obtained with the μ-GC columns. The *R_s_* values for the post structure μ-GC column between F and B, between B and T, between T and E, and between E and X were 0.63, 2.31, 0.52, and 1.24, respectively. By contrast, the *R_s_* values for the bump structure μ-GC column between F and B, between B and T, between T and E, and between E and X were 2.22, 5.19, 0.87, and 1.77, respectively, thus presenting *R_s_* values greater than those of the semi-packed-type column.

The bump structure μ-GC column showed excellent separation efficiency and performance, resulting in higher separation resolutions compared with the post structure μ-GC column.

We considered that the bump structure μ-GC column has sufficient sample capacity and separation efficiency because of the larger surface area created by the bump structures in the column channel. For the semi-packed-type μ-GC column with a high aspect ratio micro-post array embedded in the column channel, the micro-post array can be easily collapsed and distorted during the microfabrication and stationary-phase coating processes of the column. Furthermore, if the micro-post structures are excessively dense, thereby enabling an increase in the sample capacity, a massive pressure drop may occur inside the column channel, resulting in the channel clogging at the defect area and a reduction of the separation efficiency. The bump structure μ-GC column proposed in this study is mechanically robust compared with the conventional semi-packed-type μ-GC column and exhibits excellent separation performance with higher separation resolution under the same column length and separation experimental conditions.

## 4. Conclusions

This study reports a mechanically robust bump structure μ-GC column with a comparable separation performance. The μ-GC column has an internal microfluidic channel wherein the bump structures are alternately disposed to face each other on the surface of the microchannel. It was fabricated on a 2 cm × 2 cm size μ-GC chip and coated with the OV-1 thin-film stationary phase, which has a length of 1.5 m, a width of 150 μm, and a depth of 400 μm. Based on the Golay equation, the separation efficacy of the developed bump structure μ-GC column was compared with the post structure μ-GC column and commercial column. We found that the fabricated bump structure μ-GC column yielded a HETP of 0.009 cm (11,110 plates/m) at an optimal carrier gas velocity of 17 cm/s. The post structure μ-GC column and commercial GC column had the HETP values of 0.014 cm (7140 plates/m) and 0.043 cm (2330 plates/m), respectively. The developed μ-GC column showed about 1.6 times higher separation efficiency than the post structure μ-GC column in the comparison of HETP values. In addition, the plate number of the bump structure μ-GC column is comparable to the reported plate number of 4200~12,800 plates/m in other recent studies related to MEMS-based columns with similar physical dimensions. However, the developed bump structure μ-GC column is mechanically robust compared to the conventional post structure μ-GC column reported in other literatures.

The separation performance tests of the fabricated μ-GC columns were performed using the commercial FID system for the FBTEX mixture gas known as representative hazardous VOCs. The developed bump structure μ-GC column showed higher separation efficiency than the post structure μ-GC column. 

The developed column can be used in various applications requiring analyses of complex gas mixtures, such as non-invasive medical diagnoses, indoor air quality monitoring, and outdoor environmental monitoring.

## Figures and Tables

**Table 1:** Summary of the retention time (*t_r_*) and peak width at the base (*w_b_*), as obtained from the separation results for each column presented in Figure 6 and Figure 7.

Analyte	Separation Results for μ-GC Columns			
	Post Structure μ-GC Column		Bump Structure μ-GC Column	
	Retention Time, *t_r_* (min)	Peak Width, *w_b_* (min)	Retention Time, *t_r_* (min)	Peak Width, *w_b_* (min)
**Formaldehyde**	0.65	0.13	0.95	0.19
**Benzene**	0.76	0.22	1.36	0.18
**Toluene**	1.21	0.17	2.19	0.14
**Ethylbenzene**	1.32	0.25	2.32	0.16
***o*** **-Xylene**	1.68	0.33	2.63	0.19
